# Inhibition of SARS-CoV-2 3CL M^pro^ by Natural and Synthetic Inhibitors: Potential Implication for Vaccine Production Against COVID-19

**DOI:** 10.3389/fmolb.2021.640819

**Published:** 2021-04-12

**Authors:** Anwar Ullah, Kifayat Ullah

**Affiliations:** Department of Biosciences, COMSATS University Islamabad, Islamabad, Pakistan

**Keywords:** COVID-19, SARS-CoV-2, main proteinase or 3CL M^pro^, inhibition, Suramin, 2S albumin, flocculating proteins

## Abstract

COVID-19 has created a pandemic situation all over the world. It has spread in nearly every continent. Researchers all over the world are trying to produce an effective vaccine against this virus, however; no specific treatment for COVID-19 has been discovered -so far. The current work describes the inhibition study of the SARS-CoV-2 main proteinase or 3CL M^pro^ by natural and synthetic inhibitors, which include 2S albumin and flocculating protein from *Moringa oleifera* (*M. oleifera*) and Suramin. Molecular Docking study was carried out using the programs like AutoDock 4.0, HADDOCK2.4, patchdock, pardock, and firedock. The global binding energy of Suramin, 2S albumin, and flocculating proteins were −41.96, −9.12, and −14.78 kJ/mol, respectively. The docking analysis indicates that all three inhibitors bind at the junction of domains II and III. The catalytic function of 3CL M^pro^ is dependent on its dimeric form, and the flexibility of domain III is considered important for this dimerization. Our study showed that all three inhibitors reduce this flexibility and restrict their motion. The decrease in flexibility of domain III was further confirmed by analysis coming from Molecular dynamic simulation. The analysis results indicate that the temperature B-factor of the enzyme decreases tremendously when the inhibitors bind to it. This study will further explore the possibility of producing an effective treatment against COVID-19.

## Introduction

A new virus named severe acute respiratory syndrome coronavirus 2 (SARS-CoV-2) was identified in patients in China in December 2019 (Kotta et al., 2020). It spread throughout the country and world quickly and infected millions of people all over the world (Kneller et al., 2020). Till now (November 2020), 55.6 million people have been detected with this virus of which 35.86 million have been recovered and 1.34 million have died (Johns Hopkins University). The disease produced by SARS-CoV-2 is termed COVID-19 (Hussin et al., 2020; Rothan et al., 2020), which is a short name given to this disease by the World Health Organization (WHO, 2020).

The coronavirus spread through the air and physical contact among people (Graham Carlos et al., 2020; Helmy et al., 2020; Rothan and Byrareddy, 2020; Zhao et al., 2020). The usual symptoms of COVID-19 include mild fever, cough, lethargy, dyspnea (difficulties in breathing), and anosmia (loss of smell) and taste (ageusia) (Kotta et al., 2020). These symptoms usually appear after 5 days of infection by the virus (Li et al., 2020). Interestingly some of these symptoms like mild fever, cough, lethargy, and dyspnea are common among both betacoronavirus and COVID-19 (Huang C. et al., 2020); however, COVID-19 displays some distinctive clinical symptoms like sore throat, a runny nose (rhinorrhea), and sneezing (sternutation) (Lee et al., 2003; Assiri et al., 2013).

One way to prevent the COVID-19 from spreading among people is to keep a suitable distance of 1.5–2 meter as recommended by WHO (Carlos et al., 2020; Kotta et al., 2020), although a recent study has suggested that the virus can travel more than 2 m in the air (Setti et al., 2020; van Doremalen et al., 2020). The lockdown option is used in all countries of the world to achieve this social distancing and it has worked tremendously like in China (Carlos et al., 2020; Wu et al., 2020).

Currently, there is no specific treatment for COVID-19 (Barati et al., 2020), though some antiviral drugs like redeliver, oseltamivir, lopinavir/ritonavir, ganciclovir chloroquine, and hydroxychloroquine are used that can produce some relief to the patients (Costanzo et al., 2020; Kumar et al., 2020; Ledford, 2020).

The genomics and proteomics of SARS-COV2 have been described in the literature (Vandelli et al., 2020). The structure of this new virus is composed of single-stranded ribonucleic acid (RNA) and displays high sequence identity to other beta-coronavirus such as SARS-CoV and MERS-CoV (Middle East respiratory syndrome coronavirus) (Cascella et al., 2020). These viruses use a specific protein named spike (S) protein to adhere specifically to the angiotensin-converting enzyme (ACE2) on the host cell (Park et al., 2019; Turoòová et al., 2020). Besides spike glycol protein, the SARS-COV2 contains proteins like 3CL M^pro^ [also called the main proteinase (M^pro^)] and RNA-dependent RNA polymerase (RdRp) (Jeong et al., 2020).

The life cycle of SARS-COV2 begins when the virus infects the host cell through the interaction of S protein with the angiotensin I-converting enzyme-2 (ACE2) (V’kovski et al., 2020). The S protein has two subunits called S1 and S2 (Huang Y. et al., 2020), S1 it uses to attach to the N-terminal of ACE2, and the S2 subunit assists in the binding of the protein to the host membrane. This results in the binding of the virus to the membrane of the host cell. Consequently, the disruption of the membrane of the host cell occurs and endocytosis takes place (V’kovski et al., 2020). The furin proteinase and transmembrane serine proteinase 2 of the host cells cause the cleavage of S protein at the S1/S2 boundary position (V’kovski et al., 2020), which allow the entry of transmembrane serine proteinase 2-dependent entrance to the host cells (Belouzard et al., 2009; Hoffmann et al., 2020; Walls et al., 2020). The polycistronic RNA of the virus is released into the cytoplasm. The ribosomal-1 frameshifts then translates the replicase gene either into replicase polyprotein pp1a or into pp1ab (∼750 kDa, nsp1-16). This process occurs near the 3′-end of ORF 1a. This autoproteolytic cleavage results into 16 non-structural proteins (NS) by two ORF1a encoded proteinase domains (Brierley et al., 1989; Herold et al., 1993; Thiel et al., 2001, 2003; Harcourt et al., 2004; Prentice et al., 2004; Ziebuhr, 2004). The two other proteinases assisting in these proteolytic cleavages include the main proteinase M^pro^ (3CL M^pro^) and papain-like proteinase (PL^pro^) (Hegyi and Ziebuhr, 2002). The polyprotein pp1ab is cleaved by M^pro^ (Ziebuhr et al., 2000; Hegyi and Ziebuhr, 2002). The replication (production of the entire genome) or transcription (synthesis of intermittent mRNAs) is intervened by cytoplasmic enzyme complex termed replicase-transcriptase complex (Gorbalenya et al., 2006; Pasternak et al., 2006; Sawicki et al., 2007). The key proteins (structural and accessory) are translated from these transcripts; consequently the viruses are released into the cell (V’kovski et al., 2020).

The two important proteins in the life cycle of SARS-CoV-2, are the S protein and 3CL M^pro^ (Kneller et al., 2020; V’kovski et al., 2020). As discussed earlier, the S protein help the virus to binds to the host cell and to facilitate its entry to the host cell (Duan et al., 2020), while 3CL M^pro^ or the main proteinase assists in the processing of the polyproteins (Kneller et al., 2020). Owing to the main roles of these two proteins, researchers from all over the world are targeting these proteins to find out a new treatment for COVID-19 (Kotta et al., 2020). Taking this into consideration, the current work has been designed to test the efficacy of natural and synthetic inhibitors (2S albumin and flocculating proteins of *Moringa oleifera* and Suramin), against 3CL M^pro^ and discover a new treatment for this pandemic disease.

## Materials and Methods

### Atomic Structure of SARS-CoV-2 3CL M^pro^ and Ligands

The atomic coordinates of SARS-CoV-2 3CL M^pro^, Suramin, and 2S albumin were retrieved from the Protein Data Bank (PDB), with PDB IDs: 6WQF (Kneller et al., 2020), 6CE2 (SVR) (Salvador et al., 2018), and 5DOM (Ullah et al., 2015). The structure of flocculating protein was obtained as a model using the Swiss Model (Waterhouse et al., 2018). The three-dimensional atomic structure of 2S albumin from *M. oleifera* was used as a template (74% sequence identity).

### Protein and Ligand Preparation for Docking

The ligands and crystallographic water molecules were removed from the protein and the H-atoms were added. The ionization states of the atoms were kept in the ligand as mentioned in the database. The optimization of the ligand geometry was done using the AM1 method (Dewar et al., 1985). The partial charges of the ligands were calculated by AM1-BCC method (Jakalian et al., 2002). The atoms type, bond angle, dihedral, and van der Waals parameters for the ligands were assigned using the general AMBER force field (GAFF) method (Wang et al., 2004).

### Molecular Docking

The programs used for molecular docking include AutoDock 4.0 (Morris et al., 2009), pardock (Gupta et al., 2007), patchdock (Schneidman-Duhovny et al., 2005), HADDOCK2.4 (van Zundert et al., 2016), and the refinement of the docked ligands with protein was carried out using firedock (Mashiach et al., 2008). The binding affinity of the docked ligands were find out using Kdeep web server (Jiménez et al., 2018).

### Protein and Ligands Binding Interactions

The interactions (hydrogen bonds and hydrophobic contacts) between 3CL M^pro^ was determined using LigPlot (Wallace et al., 1995) from PDBsum web server (Laskowski et al., 2018).

### Molecular Dynamic Simulation

The MDMoby and MDweb programs (Hospital et al., 2012), GROMACS (Berendsen et al., 1995), AMBER16 (Case et al., 2005; Maier et al., 2015) were used for Molecular Dynamic Simulation as described previously (Ullah et al., 2019; Ullah and Masood, 2020). The all–atom–protein interaction was found out using FF14SB force field (Darden et al., 1993). The online server H + + (Anandakrishnan et al., 2012) was used for the determination of the protonation states of the amino acid side chain at pH 7.0. The neutralization of the system was carried out using Cl-. The minimization of the simulation system was carried out in order to remove the clashes in the atomic position, structural errors (bond length and bond angle). This minimization was done by executing a 500-step descent (SD) minimization, accompanied by a 2 ns position restricted MD simulation with NVT and NPT ensemble separately (Zhang et al., 2013). Subsequently, it was put in a rectangular box of TIP3P water, and extended to a minimum of 20 Å from any protein atom. The system was heated gradually from 0 to 350 K for 250 ps with a constant atom number and volume. The protein was kept with a constant force of 10 kcal/mol.Å^2^. A constant atom number, pressure, and temperature (NPT) ensemble was conducted for 500 ps to attain the equilibration step. The simulation was executed for 100 ns with a 4-fs time step. The pressure was kept at 1 atm using Nose ì-Hoover Langevin Piston algorithm (Tu et al., 1995) and the temperature was kept at 300 K, using Langevin coupling (Washio et al., 2018). The long-range electrostatic interactions were calculated using the particle-mesh Ewald (PME) method (Darden et al., 1993), by retaining the cutoff distance of Van der Waals interactions at 10 Å.

### Surface Charge Determination and Visualization

The protein and ligands were prepared for surface charge distribution using PDB2PQR (Dolinsky et al., 2007) and the charges were visualized using ABS Tools from PyMOL (DeLano, 2000).

## Results and Discussion

### The Overall Structure of SARS-CoV-2 3CL M^pro^

**FIGURE 1:**
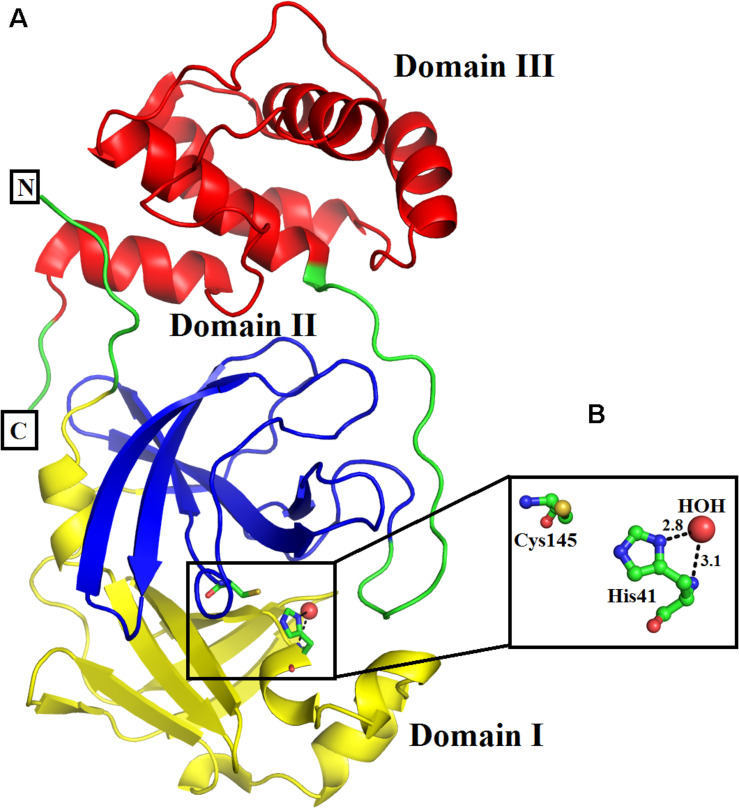
**(A)** Overall three-dimensional structure of SARS-CoV-2 3CL M^pro^. The domains I, II, and III are colored in blue, green, and red, respectively. **(B)** Active site amino acid residues of SARS-CoV-2 3CL M^pro^. The amino acid residues are shown as balls and sticks, while the catalytic water as red sphere.

The three-dimensional structure of SARS-CoV-2 3CL M^pro^ has been described by Kneller et al. (2020) with PDB ID: 6M03. The structure is composed of 306 amino acid residues and these amino acid residues fold into distinct three domains, named domains I, II, and III (Figure 1A). Domain I is composed of amino acid residues, from, Phy8-Tyr101, and has four α-helices and seven beta-strands. Domain II (amino acid residues, Lys102-Pro184) comprises seven beta strands only, whereas domain III (amino acid residues Thr201-Val303) contains five alpha-helices only. The enzyme active site is situated at the junction of domains I and II and comprises the amino acid residues His41 and Cys145 (Figure 1B), which make a dyad (Cys145-His41) instead of the triad (His47-Asp102-Ser195) as in the case of classical serine proteinases (Ullah et al., 2018). A catalytic water molecule is also bound to His41 and helps in the catalytic process of this enzyme (Figure 1B). The enzyme is active in the dimeric state and the flexibility of domain III is required for its dimerization (Kneller et al., 2020).

### Interaction Between SARS-CoV-2 3CL M^pro^ and Suramin

**FIGURE 2:**
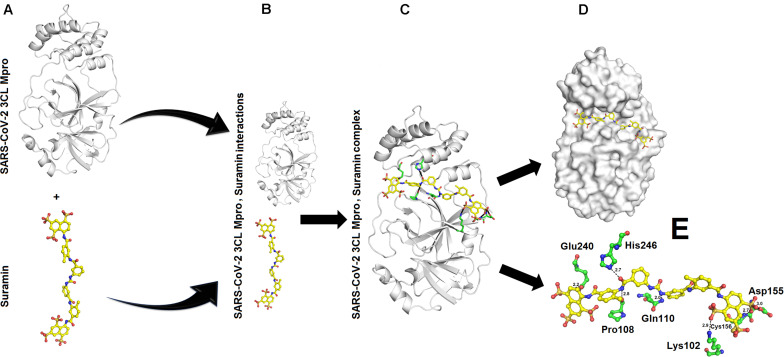
Interaction between SARS-CoV-2 3CL M^pro^ and Suramin: **(A)** Structure of SARS-CoV-2 3CL M^pro^ and Suramin **(B)** SARS-CoV-2 3CL M^pro^ and Suramin approaching each other **(C)** SARS-CoV-2 3CL M^pro^, Suramin complex **(D)** Suramin (shown as yellow sticks) residing in the cleft of SARS-CoV-2 3CL M^pro^ between domains II and III **(E)** Amino acid residues (shown as green sticks) of SARS-CoV-2 3CL M^pro^ interacting with Suramin (yellow sticks).

The binding energy calculated for interaction between SARS-CoV-2 3CL M^pro^ and Suramin was ∼−42 kcal/mol (Table 1). All the other form of bond energies are listed in Tables 1, 2. Suramin binding site is between the two domains (Domains II and III) of SARS-CoV-2 3CL M^pro^ (Figures 2A–C). The amino acid residues of SARS-CoV-2 3CL M^pro^ that interact with Suramin include Lys102, Pro108, Gln110, Asp155, Glu240, and His246 (Figures 1D,E). The Kdeep results indicate that both equilibrium dissociation constant (pKd) and Gibb’s free energy (ΔG) are large (Table 3), which further confirmed the binding between SARS-CoV-2 3CL M^pro^ and the three ligands (Suramin, 2S albumin and Flocculating protein). The LigPlot analysis indicates a total of seven hydrogen bonds and 263 non-bonded or hydrophobic interactions between SARS-CoV-2 3CL M^pro^ and Suramin (Supplementary Figure 3 and Supplementary Table 1).

**TABLE 1:** Output data from FireDock server.

**Ligands/inhibitor**	**Global binding energy (kj/mol)**	**Attractive VdW**	**Repulsive VdW**	**ACE**	**HB**
Suramin	–41.96	–26.83	15.40	–12.34	0.00
2S albumin	–9.12	–29.45	17.09	0.83	–0.63
Flocculating protein	–14.78	–25.14	15.39	0.30	–1.08

*Global binding energy, attractive VdW (van der Walls forces), Repulsive VdW, ACE (atomic contact energy) and HB (contribution of the hydrogen bonds to global energy) for the interaction between SARS-CoV-2 3CL M^pro^, Suramin, 2S albumin and flocculating protein).*

**TABLE 2:** HADDOCK score and various form of bond energies for docking among SARS-CoV-2 3CL M^pro^, Suramin, 2S albumin and flocculating protein.

**Protin and ligand complex**	**Suramin**	**2S albumin**	**Flocculating protein**
HADDOCK score	−49.03.1	−72.85.9	−71.28.9
Cluster size	81	6	10
RMSD from the overall lowest-energy structure	1.40.4	0.90.1	0.70.4
Van der Waals energy	−24.05.2	−30.63.0	−27.74.0
Electrostatic energy	−190.343.8	−168.810.8	−118.810.0
Desolvation energy	4.92.3	−7.34.3	−7.55.7
Restraints violation energy	0.71.13	39.02.89	37.325.74
Buried Surface Area	0.71.13	1199.938.6	1166.543.2
Z-Score	−1.8	−1.7	−2.1

**TABLE 3:** Binding affinity results from K_DEEP:_ M.wt., Molecular weight; pKd, equilibrium dissociation constant (pKd, -log (Kd); ΔG, Gibbs free energy.

**Ligands**	**M.wt (g/mol)**	**pKd (std.)**	**ΔG (Kcal/mol (std.)**	**Lig. Efficiency (Kcal/mol)**
Suramin	1427.94	12.75 (2.03)	−17.21 (−2.75)	−0.19
2S albumin	14271.17	98.04 (96.61)	−93.31 (−92.11)	−0.27
Flocculating proteins	6282.17	56.05 (55.72)	−75.67 (−75.54)	−0.17

Suramin is a drug that is used to treat African sleeping sickness and river blindness (Lima et al., 2009). Suramin has been shown to inhibit Human α-thrombin (Lima et al., 2009), snake venom phospholipases A2 (Salvador et al., 2018), snake venom serine proteinases (Ullah et al., 2018), severe Fever with thrombocytopenia syndrome virus nucleocapsid protein (Jiao et al., 2013), murine Norovirus RNA-dependent RNA polymerase (Mastrangelo et al., 2012), and Leishmania mexicana pyruvate kinase (Morgan et al., 2011). In most of these cases, the Suramin binds toward the C-terminal of the proteins and restrict the motion of the C-terminal (Lima et al., 2009; Ullah et al., 2018). In the current study, Suramin binds toward the N-terminal of SARS-CoV-2 3CL M^pro^ (Figures 2A–E).

### Interaction Between SARS-CoV-2 3CL M^pro^, 2S Albumin and Flocculating Protein

**FIGURE 3:**
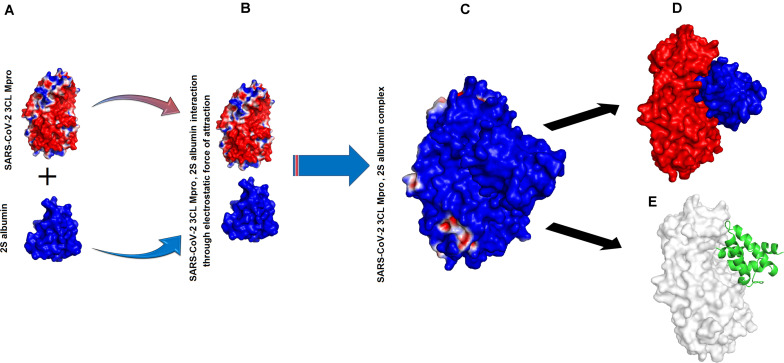
Interaction between SARS-CoV-2 3CL M^pro^ and 2S albumin: **(A)** Surface charge representation of SARS-CoV-2 3CL M^pro^ and 2S albumin **(B)** SARS-CoV-2 3CL M^pro^ and 2S albumin approaching each other **(C)** SARS-CoV-2 3CL M^pro^, 2S albumin complex **(D)** 2S albumin (shown as blue surface) residing in the cleft of SARS-CoV-2 3CL M^pro^ between domains II and III (red colored) **(E)** 2S albumin (shown as green cartoon) interacting with SARS-CoV-2 3CL M^pro^ (shown as white surface).

**FIGURE 4:**
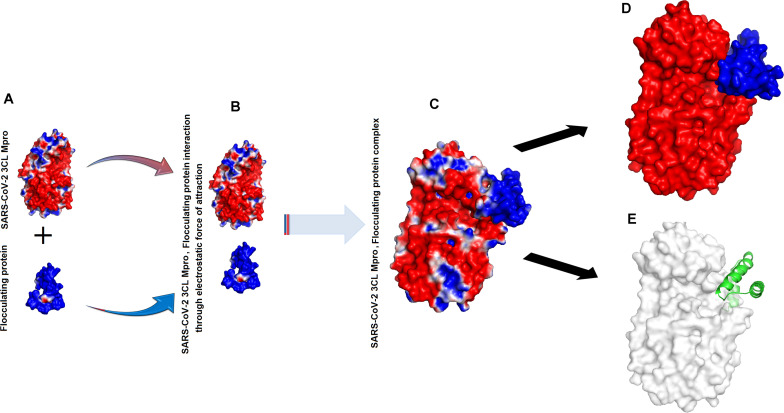
Interaction between SARS-CoV-2 3CL M^pro^ and flocculating protein: **(A)** Surface charge representation of SARS-CoV-2 3CL M^pro^ and flocculating protein **(B)** SARS-CoV-2 3CL M^pro^ and flocculating protein approaching each other **(C)** SARS-CoV-2 3CL M^pro^, flocculating protein complex **(D)** flocculating protein (shown as blue surface) residing in the cleft of SARS-CoV-2 3CL M^pro^ between domains II and III (red colored) **(E)** flocculating protein (shown as green cartoon) interacting with SARS-CoV-2 3CL M^pro^ (shown as white surface).

The binding energies for SARS-CoV-2 3CL M^pro^, 2S albumin and flocculating protein were ∼−9.12 and ∼−15 kJ/mol, respectively (Table 1). The other form of bond energies come from docking as indicated in Tables 1, 2. The amino acid residues involved in these interactions, include S139, T139, G302, Q299 (SARS-CoV-2 3CL M^pro^), R143, Q97 (2S albumin) and Q15, and Q38 (Flocculating protein). The interactions between SARS-CoV-2 3CL M^pro^, 2S albumin, and flocculating protein are largely electrostatic (Figures 3A–E and 4A–E). In both cases, the ligands binding site is between the two domains (Domains II and III) of SARS-CoV-2 3CL M^pro^. The LigPlot analysis shows a total of three hydrogen bonds between SARS-CoV-2 3CL M^pro^ and both 2S albumin and flocculation protein, while the number of hydrophobic interactions were 130 and 152 for 2S albumin and flocculating protein, respectively (Supplementary Figures 4,5).

### Molecular Dynamic Simulation Analysis for SARS-CoV-2 3CL M^pro^ Alone and With the Ligands

The MD simulation analysis indicates that the flexibility of SARS-CoV-2 3CL M^pro^ decreases tremendously when the ligands bind to it (Supplementary Figure 1). For Suramin as an inhibitor, the fluctuation increases a little bit (temperature B-factor increases from 14 to 16) (Supplementary Figure 1 and Figures 2A–D), while in the case of 2S albumin and flocculating proteins the fluctuation decreases (temperature B-factor decreases from 12 to 10, respectively (Supplementary Figure 1, Figures 3A–D and 4A–D). The RMSD vs. time graph indicates that the interaction between SARS-CoV-2 3CL M^pro^ and the three ligands was stable throughout the simulation process (Supplementary Figure 6). Suramin can make 1–5 hydrogen bonds, while both 2S albumin and flocculating protein can make 2–5 hydrogen bonds according to 100 ns MD simulation analysis (Supplementary Figure 7).

The flexibility analysis from PyMOL also indicates that all the ligands decrease the flexibility of SARS-CoV-2 3CL M^pro^ upon binding (Supplementary Figures 2A–D).

### Inhibition Study of SARS-COV-2 3CL M^pro^ by Other Researchers

Teli et al. (2021), have screened ten compounds namely, Procyanidin A3, Rutin, Solanine, Procyanidin A4, Procyanidin B4, Hypericin, Quercetagetin, Procyanidin, and Astragalin for inhibition of SARS-COV-2 3CL M^pro^. In that study they have shown that most of these compounds binds in the active site cavity of SARS-COV-2 3CL M^pro^ (Teli et al., 2021). Chourasia et al. (2020) have used a potential peptide (with amino acid sequence, KFVPKQPNMIL) from soy cheese for effective Inhibition of SARS-CoV-2 Main Protease and S1 Glycoprotein (Chourasia et al., 2020). This peptide binds specifically to the amino acid residues that are important for the host cell entry and multiplication (3CL^pro^) of SARS-CoV-2. Abdusalam and Murugaiyah (2020) have used ZINC database to identify zinc containing compounds as inhibitors of SARS-COV-2 3CL M^pro^, and have encountered four active zinc compounds (ZINC32960814, ZINC12006217, ZINC03231196, and ZINC33173588) which shows high binding affinity for 3CL^pro^ pocket (Abdusalam and Murugaiyah, 2020). Vincent et al. (2020) have used Compounds From Kabasura Kudineer on SARS-CoV-2 3CL^pro^ and have shown that Acetoside, Luteolin 7, rutinoside, rutin, Chebulagic acid, Syrigaresinol, Acanthoside, Violanthin, Andrographidine C, myricetin, Gingerenone -A, Tinosporinone, Geraniol, Nootkatone, Asarianin, and Gamma sitosterol are the natural compounds in Kabasura Kudineer extracts, which can used as effective inhibitors against SARS-CoV-2 3CL^pro^ (Vincent et al., 2020).

## Conclusion

• The inhibition of 3CL M^pro^ by natural (2S albumin and flocculating protein from *M. oleifera*) and synthetic inhibitor (Suramin) was demonstrated in this study.
• The interaction between 3CL M^pro^ and the inhibitors are largely through electrostatic force of attraction and with the interactions of amino acid residues from both sides.
• All the three inhibitors bind between domain II and III (3CL M^pro^ amino acid residues, Lys102, Pro108, Gln110, Asp155, Glu240, and His246, with Suramin and S139, T139, G302, Q299 with 2S albumin and flocculating protein. These interactions restrict the moment in domain III, which is important for dimerization and further for the function of SARS-COV2 3CL M^pro^.
• Here we proposed that these inhibitors will inhibit 3CL M^pro^ by preventing this enzyme from dimerization.
• The current study will lead to the production of a new vaccine against COVID-19.

## Data Availability Statement

The original contributions presented in the study are included in the article/Supplementary Material, further inquiries can be directed to the corresponding author.

## Author Contributions

AU designed the project and reviewed the manuscript. KU drafted and proofread the manuscript, and did English language corrections in the manuscript revision stage. Both authors contributed to the article and approved the submitted version.

## Conflict of Interest

The authors declare that the research was conducted in the absence of any commercial or financial relationships that could be construed as a potential conflict of interest.

## Supplementary Material

Supplementary Figure 1 Molecular dynamic simulation of SARS-CoV-2 3CL Mpro and bound ligands. SARS-CoV-2 3CL Mpro, **(A)** RMSD values per residue **(B)** Trajectory RMSD **(C)** Radius of Gyration **(D)** B-factor per residue. SARS-CoV-2 3CL Mpro in the presence of Suramin, **(A)** RMSD values per residue **(B)** Trajectory RMSD **(C)** Radius of Gyration **(D)** B-factor per residue. SARS-CoV-2 3CL Mpro in the presence of 2S albumin, **(A)** RMSD values per residue **(B)** Trajectory RMSD **(C)** Radius of Gyration **(D)** B-factor per residue. SARS-CoV-2 3CL Mpro in the presence of flocculating, **(A)** RMSD values per residue **(B)** Trajectory RMSD **(C)** Radius of Gyration **(D)** B-factor per residue.

Supplementary Figure 2 Temperature B-factor of SARS-CoV-2 3CL Mpro and bound ligands.**(A)** SARS-CoV-2 3CL Mpro alone **(B)** SARS-CoV-2 3CL Mpro with Suramin **(C)** SARS-CoV-2 3CL Mpro with 2S albumin **(D)** SARS-CoV-2 3CL Mpro with flocculating protein.

Supplementary Video 1 Moments in the domains of SARS-CoV-2 3CL Mpro.

## References

[B1] Abdusalam A. A. A. Murugaiyah V. (2020). Identification of Potential Inhibitors of 3CL Protease of SARS-CoV-2 From ZINC Database by Molecular Docking-Based Virtual Screening. *Front. Mol. Biosci.* 7:603037. 10.3389/fmolb.2020.603037 33392261 PMC7773842

[B2] Anandakrishnan R. Aguilar B. Onufriev A. V. (2012). H++ 3.0: automating pK prediction and the preparation of biomolecular structures for atomistic molecular modeling and simulation. *Nucleic Acids Res.* 40 W537–W541. 22570416 10.1093/nar/gks375 PMC3394296

[B3] Assiri A. Al-Tawfiq J. A. Al-Rabeeah A. A. (2013). Epidemiological, demographic, and clinical characteristics of 47 cases of Middle East respiratory syndrome coronavirus disease from Saudi Arabia: a descriptive study. *Lancet Infect. Dis.* 13 752–761. 10.1016/s1473-3099(13)70204-4 23891402 PMC7185445

[B4] Barati F. Pouresmaieli M. Ekrami E. Asghari S. Ziarani F. R. Mamoudifard M. (2020). Potential Drugs and Remedies for the Treatment of COVID-19: a Critical Review. *Biol Proced Online* 22:15. 10.1186/s12575-020-00129-1 32754003 PMC7377207

[B5] Belouzard S. Chu V. C. Whittaker G. R. (2009). Activation of the SARS coronavirus spike protein via sequential proteolytic cleavage at two distinct sites. *Proc. Natl. Acad. Sci. U. S. A.* 106 5871–5876. 10.1073/pnas.0809524106 19321428 PMC2660061

[B6] Berendsen H. J. C. van der Spoel D. van Drunen R. (1995). GROMACS: A message-passing parallel molecular dynamics implementation. *Comput. Phys. Commun.* 91 43–56. 10.1016/0010-4655(95)00042-e

[B7] Brierley I. Digard P. Inglis S. C. (1989). Characterizatio,.n of an efficient coronavirus ribosomal frameshifting signal: requirement for an RNA pseudoknot. *Cell* 57 537–547. 10.1016/0092-8674(89)90124-4 2720781 PMC7133225

[B8] Carlos W. G. Dela Cruz C. S. Cao B. Pasnick S. Jamil S. (2020). Novel wuhan (2019-nCoV) coronavirus. *Am. J. Respir. Crit. Care Med.* 201 7–8. 10.1164/rccm.2014P7 32004066

[B9] Cascella M. Rajnik M. Cuomo A. Dulebohn S. C. DiNapoli R. (2020). *Features, evaluation and treatment coronavirus(COVID-19).* Treasure Island: StatPearls Publishing. 32150360

[B10] Case D. A. Cheatham T. E. Darden T. Gohlke H. Luo R. Merz K. M. (2005). The Amber biomolecular simulation programs. *J. Computat. Chem.* 26 1668–1688. 10.1002/jcc.20290 16200636 PMC1989667

[B11] Chourasia R. Padhi S. Chiring Phukon L. Abedin M. M. Singh S. P. Rai A. K. (2020). A Potential Peptide From Soy Cheese Produced Using *Lactobacillus delbrueckii* WS4 for Effective Inhibition of SARS-CoV-2 Main Protease and S1 Glycoprotein. *Front. Mol. Biosci.* 7:601753. 10.3389/fmolb.2020.601753 33363209 PMC7759660

[B12] Costanzo M. De Giglio M. A. R. Roviello G. N. (2020). SARS-CoV-2: Recent Reports on Antiviral Therapies Based on Lopinavir/Ritonavir, Darunavir/Umifenovir, Hydroxychloroquine, Remdesivir, Favipiravir and other Drugs for the Treatment of the New Coronavirus. *Curr Med Chem.* 27 4536–4541. 10.2174/0929867327666200416131117 32297571

[B13] Darden T. York D. Pedersen L. (1993). Particle mesh Ewald: an N log (N) method for Ewald sums in large systems. *J. Chem. Phys.* 98 10089–10092. 10.1063/1.464397

[B14] DeLano. (2000). *The PyMOL Molecular Graphics System, Version 2.0 Schrödinger, LLC.*

[B15] Dewar M. J. Zoebisch E. G. Healy E. F. Stewart J. J. (1985). Development and use of quantum mechanical molecular models. 76. AM1: a new general purpose quantum mechanical molecular model. *J. Am. Chem. Soc.* 107 3902–3909. 10.1021/ja00299a024

[B16] Dolinsky T. J. Czodrowski P. Li H. Nielsen J. E. Jensen J. H. Klebe G. (2007). PDB2PQR: expanding and upgrading automated preparation of biomolecular structures for molecular simulations. *Nucleic Acids Res.* 35 W522–W525. 10.1093/nar/gkm276 17488841 PMC1933214

[B17] Duan L. Zheng Q. Zhang H. Niu Y. Lou Y. Wang H. (2020). The SARS-CoV-2 Spike Glycoprotein Biosynthesis, Structure, Function, and Antigenicity: Implications for the Design of Spike-Based Vaccine Immunogens. *Front Immunol.* 7:576622. 10.3389/fimmu.2020.576622 33117378 PMC7575906

[B18] Gorbalenya A. E. Enjuanes L. Ziebuhr J. Snijder E. J. (2006). Nidovirales: evolving the largest RNA virus genome. *Virus Res.* 117 17–37. 10.1016/j.virusres.2006.01.017 16503362 PMC7114179

[B19] Graham Carlos W. Dela Cruz C. S. Cao B. Pasnick S. Jamil S. (2020). Novel Wuhan (2019-NCoV) coronavirus. *Am. J. Respir. Crit. Care Med.* 201 7–8. 32004066 10.1164/rccm.2014P7

[B20] Gupta A. Gandhimathi A. Sharma P. Jayaram B. (2007). ParDOCK: An All Atom Energy Based Monte Carlo Docking Protocol for Protein-Ligand Complexes. *Protein Peptide Lett.* 14 632–646. 10.2174/092986607781483831 17897088

[B21] Harcourt B. H. Jukneliene D. Kanjanahaluethai A. Bechill J. Severson K. M. Smith C. M. (2004). Identification of severe acute respiratory syndrome coronavirus replicase products and characterization of papain-like protease activity. *J. Virol.* 78 13600–13612. 10.1128/JVI.78.24.13600-13612.2004 15564471 PMC533933

[B22] Hegyi A. Ziebuhr J. (2002). Conservation of substrate specificities among coronavirus main proteases. *J. Gen. Virol.* 83(Pt 3), 595–599. 10.1099/0022-1317-83-3-595 11842254

[B23] Helmy Y. A. Fawzy M. Elaswad A. Sobieh A. Kenney S. P. Shehata A. A. (2020). The COVID-19 Pandemic: A Comprehensive Review of Taxonomy, Genetics, Epidemiology, Diagnosis, Treatment, and Control. *J. Clin. Med.* 9:1225. 10.3390/jcm9041225 32344679 PMC7230578

[B24] Herold J. Raabe T. Schelle-Prinz B. Siddell S. G. (1993). Nucleotide sequence of the human coronavirus 229E RNA polymerase locus. *Virology* 195 680–691. 10.1006/viro.1993.1419 8337838 PMC7131648

[B25] Hoffmann M. Kleine-Weber H. Schroeder S. Krüger N. Herrler T. Erichsen S. (2020). SARS-CoV-2 cell entry depends on ACE2 and TMPRSS2 and is blocked by a clinically proven protease inhibitor. *Cell* 181 271.e–280.e. 10.1016/j.cell.2020.02.052 32142651 PMC7102627

[B26] Hospital P. A. Andrio C. Fenollosa D. Cicin-Sain M. Orozco J. L. (2012). MDWeb and MDMoby: an integrated web-based platform for molecular dynamics simulations. *Bioinformatics* 28 1278–1279. 10.1093/bioinformatics/bts139 22437851

[B27] Huang C. Wang Y. Li X. Ren L. Zhao J. Hu Y. (2020). Clinical features of patients infected with 2019 novel coronavirus in Wuhan, China. *Lancet* 395 497–506. 10.1016/S0140-6736(20)30183-5 31986264 PMC7159299

[B28] Huang Y. Yang C. Xu X. F. Xu W. Liu S. W. (2020). Structural and functional properties of SARS-CoV-2 spike protein: potential antivirus drug development for COVID-19. *Acta Pharmacol Sin.* 41 1141–1149. 10.1038/s41401-020-0485-4 32747721 PMC7396720

[B29] Hussin A. R. Siddappa N. Byrareddy B. C. D. (2020). The epidemiology and pathogenesis of coronavirus disease (COVID-19) outbreak. *J. Autoimmunity* 109:102433. 10.1016/j.jaut.2020.102433 32113704 PMC7127067

[B30] Jakalian A. Jack D. B. Bayly C. I. (2002). Fast, efficient generation of high-quality atomic charges. AM1-BCC model: II. Parameterization and validation. *J. Comput. Chem.* 23 1623–1641. 10.1002/jcc.10128 12395429

[B31] Jeong G. U. Song H. Yoon G. Y. Kim D. Kwon Y. C. (2020). Therapeutic Strategies Against COVID-19 and Structural Characterization of SARS-CoV-2: A Review. *Front. Microbiol.* 11:1723. 10.3389/fmicb.2020.01723 32765482 PMC7381222

[B32] Jiao L. Ouyang S. Liang M. Niu F. Shaw N. Wu W. (2013). Structure of severe fever with thrombocytopenia syndrome virus nucleocapsid protein in complex with suramin reveals therapeutic potential. *J. Virol.* 87 6829–6839. 10.1128/JVI.00672-13 23576501 PMC3676114

[B33] Jiménez J. Škaliè M. Martínez-Rosell G. De Fabritiis G. (2018). K_DEEP_: Protein-Ligand Absolute Binding Affinity Prediction via 3D-Convolutional Neural Networks. *J. Chem. Inf. Model.* 58 287–296. 10.1021/acs.jcim.7b00650 29309725

[B34] Kneller D. W. Phillips G. O’Neill H. M. Jedrzejczak R. Stols L. Langan P. (2020). Structural plasticity of SARS-CoV-2 3CL M^pro^ active site cavity revealed by room temperature X-ray crystallography. *Nat. Commun.* 11:3202. 10.1038/s41467-020-16954-7 32581217 PMC7314768

[B35] Kotta S. Aldawsari H. M. Badr-Eldin S. M. Alhakamy N. A. Md S. Nair A. B. (2020). Combating the Pandemic COVID-19: Clinical Trials. Therapies and Perspectives. *Front. Mol. Biosci.* 7:606393. 10.3389/fmolb.2020.606393 33282914 PMC7705351

[B36] Kumar S. Zhi K. Mukherji A. Gerth K. (2020). Repurposing Antiviral Protease Inhibitors Using Extracellular Vesicles for Potential Therapy of COVID-19. *Viruses* 12:486. 10.3390/v12050486 32357553 PMC7290948

[B37] Laskowski R. A. Jabłońska J. Pravda L. Vařeková R. S. Thornton J. M. (2018). PDBsum: Structural summaries of PDB entries. *Protein Sci.* 27 129–134. 10.1002/pro.3289 28875543 PMC5734310

[B38] Ledford H. (2020). The race to make COVID antibody therapies cheaper and more potent. *Nature.* 587:18. 10.1038/d41586-020-02965-3 33097846

[B39] Lee N. Hui D. Wu A. Chan P. Cameron P. Joynt G. M. (2003). A major outbreak of severe acute respiratory syndrome in Hong Kong. *N. Engl. J. Med.* 348 1986–1994. 12682352 10.1056/NEJMoa030685

[B40] Li Q. Guan X. Wu P. Wang X. Zhou L. Tong Y. (2020). Early transmission dynamics in Wuhan, China, of novel coronavirus-infected pneumonia. *N. Engl. J. Med.* 382 1199–1207. 31995857 10.1056/NEJMoa2001316 PMC7121484

[B41] Lima L. M. Becker C. F. Giesel G. M. Marques A. F. Cargnelutti M. T. de Oliveira Neto M. (2009). Structural and thermodynamic analysis of thrombin:suramin interaction in solution and crystal phases. *Biochim. Biophys. Acta* 1794 873–881. 10.1016/j.bbapap.2009.03.011 19332154

[B42] Maier J. A. Martinez C. Kasavajhala K. Wickstrom L. Hauser K. E. Simmerling C. (2015). ff14SB: improving the accuracy of protein side chain and backbone parameters from ff99SB. *J. Chem. Theory Comput.* 11 3696–3713. 10.1021/acs.jctc.5b00255 26574453 PMC4821407

[B43] Mashiach E. Schneidman-Duhovny D. Andrusier N. Nussinov R. Wolfson H. J. (2008). FireDock: a web server for fast interaction refinement in molecular docking. *Nucleic Acids Res.* 36 W229–W232. 18424796 10.1093/nar/gkn186 PMC2447790

[B44] Mastrangelo E. Pezzullo M. Tarantino D. Petazzi R. Germani F. Kramer D. (2012). Structure-based inhibition of Norovirus RNA-dependent RNA polymerases. *J. Mol. Biol.* 419 198–210. 10.1016/j.jmb.2012.03.008 22446684

[B45] Morgan H. P. McNae I. W. Nowicki M. W. Zhong W. Michels P. A. Auld D. S. (2011). The trypanocidal drug suramin and other trypan blue mimetics are inhibitors of pyruvate kinases and bind to the adenosine site. *J. Biol. Chem.* 286 31232–31240. 10.1074/jbc.M110.212613 21733839 PMC3173065

[B46] Morris G. M. Huey R. Lindstrom W. Sanner M. F. Belew R. K. Goodsell D. S. (2009). Autodock4 and AutoDockTools4: automated docking with selective receptor flexiblity. *J. Comput. Chem.* 2009 2785–2791. 10.1002/jcc.21256 19399780 PMC2760638

[B47] Park Y.-J. Walls A. C. Wang Z. Sauer M. M. Li W. Tortorici M. A. (2019). Structures of MERS-CoV spike glycoprotein in complex withsialoside attachment receptors. *Nat. Struct. Mol. Biol.* 26 1151–1157. 10.1038/s41594-019-0334-7 31792450 PMC7097669

[B48] Pasternak A. O. Spaan W. J. M. Snijder E. J. (2006). Nidovirus transcription: how to make sense.? *J. Gen. Virol.* 87(Pt 6), 1403–1421. 10.1099/vir.0.81611-0 16690906

[B49] Prentice E. McAuliffe J. Lu X. Subbarao K. Denison M. R. (2004). Identification and characterization of severe acute respiratory syndrome coronavirus replicase proteins. *J. Virol.* 78 9977–9986. 10.1128/JVI.78.18.9977-9986.2004 15331731 PMC514967

[B50] Rothan H. A. Byrareddy S. N. (2020). The epidemiology and pathogenesis of coronavirus disease (COVID-19) outbreak. *J. Autoimmun.* 109:102433. 10.1016/j.jaut.2020.102433 PMC7127067 32113704

[B51] Rothan H. A. Acharya A. Reid S. P. Kumar M. Byrareddy S. N. (2020). Molecular aspects of COVID-19 differential pathogenesis. *Pathogens* 9:538. 10.3390/pathogens9070538 32640525 PMC7400297

[B52] Salvador G. H. M. Dreyer T. R. Gomes A. A. S. Cavalcante W. L. G. Dos Santos J. I. Gandin C. A. (2018). Structural and functional characterization of suramin-bound MjTX-I from Bothrops moojeni suggests a particular myotoxic mechanism. *Sci. Rep.* 8:10317. 10.1038/s41598-018-28584-7 29985425 PMC6037679

[B53] Sawicki S. G. Sawicki D. L. Siddell S. G. (2007). A contemporary view of coronavirus transcription. *J. Virol.* 81 20–29. 10.1128/JVI.01358-06 16928755 PMC1797243

[B54] Schneidman-Duhovny D. Inbar Y. Nussinov R. Wolfson H. J. (2005). PatchDock and SymmDock: servers for rigid and symmetric docking. *Nuclic Acids Res.* 33 W363–W367. 10.1093/nar/gki481 PMC1160241 15980490

[B55] Setti L. Passarini F. De Gennaro G. Barbieri P. Perrone M. G. Borelli M. (2020). Airborne Transmission Route of COVID-19: Why 2 Meters/6 Feet of Inter-Personal Distance Could Not Be Enough. *Int. J. Environ. Res. Public Health* 17:2932. 10.3390/ijerph17082932 32340347 PMC7215485

[B56] Teli D. M. Shah M. B. Chhabria M. T. (2021). *In silico* Screening of Natural Compounds as Potential Inhibitors of SARS-CoV-2 Main Protease and Spike RBD: Targets for COVID-19. *Front. Mol. Biosci.* 7:599079. 10.3389/fmolb.2020.599079 33542917 PMC7852456

[B57] Thiel V. Herold J. Schelle B. Siddell S. G. (2001). Infectious RNA transcribed in vitro from a cDNA copy of the human coronavirus genome cloned in vaccinia virus. *J. Gen. Virol.* 82(Pt 6), 1273–1281. 10.1099/0022-1317-82-6-1273 11369870

[B58] Thiel V. Ivanov K. A. Putics Á Hertzig T. Schelle B. Bayer S. (2003). Mechanisms and enzymes involved in SARS coronavirus genome expression. *J Gen Virol.* 84(Pt 9), 2305–2315. 10.1099/vir.0.19424-0 12917450

[B59] Tu K. C. Tobias D. J. Klein M. L. (1995). Constant-pressure andtemperature molecular-dynamics simulations of crystals of the lecithinfragments—glycerolphophorylcholine and dilauroylglycerol.J. *Phys.Chem.* 99 10035–10042. 10.1021/j100024a053

[B60] Turoòová B. Sikora M. Schürmann C. Hagen W. J. H. Welsch S. Blanc F. E. C. (2020). In situ structural analysis of SARS-CoV-2 spike reveals flexibility mediated by three hinges. *Science* 370 203–208. 10.1126/science.abd5223 32817270 PMC7665311

[B61] Ullah A. Masood R. (2020). The Sequence and Three-Dimensional Structure Characterization of Snake Venom Phospholipases B. *Front. Mol. Biosci.* 7:175. 10.3389/fmolb.2020.00175 32850964 PMC7419708

[B62] Ullah A. Mariutti R. B. Masood R. Caruso I. P. Costa G. H. Santos C. R. (2015). Crystal structure of mature 2S albumin from Moringa oleifera seeds. *Biochem. Biophys. Res. Commun.* 468 365–371. 10.1016/j.bbrc.2015.10.087 26505799

[B63] Ullah A. Masood R. Ali I. Ullah K. Ali H. Akbar H. (2018). Thrombin-like enzymes from snake venom: Structural characterization and mechanism of action. *Int. J. Biol. Macromol.* 114 788–811. 10.1016/j.ijbiomac.2018.03.164 29604354

[B64] Ullah A. Ullah K. Ali H. Betzel C. Ur Rehman S. (2019). The Sequence and a Three-Dimensional Structural Analysis Reveal Substrate Specificity Among Snake Venom Phosphodiesterases. *Toxins* 11 625. 10.3390/toxins11110625 31661911 PMC6891707

[B65] van Doremalen N. Bushmaker T. Morris D. H. Holbrook M. G. Gamble A. Williamson B. N. (2020). Aerosol and surface stability of SARS-CoV-2 as compared with SARS-CoV-1. *N. Engl. J. Med.* 382 1564–1567. 32182409 10.1056/NEJMc2004973 PMC7121658

[B66] van Zundert G. C. P. Rodrigues J. P. G. L. M. Trellet M. Schmitz C. Kastritis P. L. Karaca E. (2016). The HADDOCK2.2 Web Server: User-Friendly Integrative Modeling of Biomolecular Complexes. *J. Mol. Biol.* 428 720–725. 10.1016/j.jmb.2015.09.014 26410586

[B67] Vandelli A. Monti M. Milanetti E. Armaos A. Rupert J. Zacco E. (2020). Structural analysis of SARS-CoV-2 genome and predictions of the human interactome. *Nucleic Acids Res.* 48 11270–11283. 10.1093/nar/gkaa864 33068416 PMC7672441

[B68] Vincent S. Arokiyaraj S. Saravanan M. Dhanraj M. (2020). Molecular Docking Studies on the Anti-viral Effects of Compounds From Kabasura Kudineer on SARS-CoV-2 3CL^pro^. *Front. Mol. Biosci.* 7:613401. 10.3389/fmolb.2020.613401 33425994 PMC7785853

[B69] V’kovski P. Kratzel A. Steiner S. Stalder H. Thiel V. (2020). Coronavirus biology and replication: implications for SARS-CoV-2. *Nat. Rev. Microbiol.* 2020 1–16. 10.1038/s41579-020-00468-6 33116300 PMC7592455

[B70] Wallace A. C. Laskowski R. A. Thornton J. M. (1995). LIGPLOT: a program to generate schematic diagrams of protein-ligand interactions. *Protein Eng.* 8 127–134. 10.1093/protein/8.2.127 7630882

[B71] Walls A. C. Park Y.-J. Tortorici M. A. Wall A. McGuire A. T. Veesler D. (2020). Structure, function, and antigenicity of the SARS-CoV-2 spike glycoprotein. *Cell* 181 281.e–292.e. 10.1016/j.cell.2020.02.058 32155444 PMC7102599

[B72] Wang J. Wolf R. M. Kollman P. A. Case D. A. (2004). Development and testing of a general AMBER force field. *J. Computat. Chem.* 25 1157–1174. 10.1002/jcc.20035 15116359

[B73] Washio T. Sugiura S. Kanada R. Okada J.-I. Hisada T. (2018). Coupling Langevin Dynamics With Continuum Mechanics: Exposing the Role of Sarcomere Stretch Activation Mechanisms to Cardiac Function. *Front. Physiol*. 9:333. 10.3389/fphys.2018.00333 29681861 PMC5898180

[B74] Waterhouse A. Bertoni M. Bienert S. Studer G. Tauriello G. Gumienny R. (2018). SWISS-MODEL: homology modelling of protein structures and complexes. *Nucleic Acids Res.* 46 W296–W303. 29788355 10.1093/nar/gky427 PMC6030848

[B75] WHO. (2020). *WHO/Europe | Coronavirus disease (COVID-19) outbreak - WHO announces COVID-19 outbreak a pandemic.* Available online at: https://www.euro.who.int/en/health-topics/health-emergencies/coronavirus-covid-19# (accessed date November 28, 2020)

[B76] Wu P. Hao X. Lau E. H. Y. Wong J. Y. Leung K. S. M. Wu J. T. (2020). *Real-time tentative assessment of the epidemiological characteristics of novel coronavirus infections in Wuhan, China, as at 22 January 2020, Euro Surveill. 25^∗^.* 10.2807/1560-7917.ES.2020.25.3.2000044 PMC6988272 31992388

[B77] Zhang D. Chen C. F. Zhao B. B. Gong L. L. Jin W. J. Liu J. J. (2013). novel antibody humanization method based on epitopes scanning and molecular dynamics simulation. *PLoS One* 8:e80636. 10.1371/journal.pone.0080636 24278299 PMC3836750

[B78] Zhao S. Lin Q. Ran J. Musa S. S. Yang G. Wang W. (2020). Preliminary estimation of the basic reproduction number of novel coronavirus (2019-nCoV) in China, from 2019 to 2020: a data-driven analysis in the early phase of the outbreak. *Int. J. Infect. Dis.* 92 214–217. 10.1016/j.ijid.2020.01.05 32007643 PMC7110798

[B79] Ziebuhr J. (2004). Molecular biology of severe acute respiratory syndrome coronavirus. *Curr. Opin. Microbiol.* 7 412–419. 10.1016/j.mib.2004.06.007 15358261 PMC7108451

[B80] Ziebuhr J. Snijder E. J. Gorbalenya A. E. (2000). Virus-encoded proteinases and proteolytic processing in the Nidovirales. *J. Gen. Virol.* 81(Pt 4), 853–879. 10.1099/0022-1317-81-4-853 10725411

