# Supplementary material for: Inhibition of SARS-CoV-2 3CL Mpro by Natural and Synthetic Inhibitors: Potential Implication for Vaccine Production Against COVID-19
Source: Front Mol Biosci. 2021 Apr 12;8:640819. doi: 10.3389/fmolb.2021.640819 (PMC8072276; doi:10.3389/fmolb.2021.640819)
Supplement: Supplementary Figure 1 — Molecular dynamic simulation of SARS-CoV-2 3CL Mpro and bound ligands. SARS-CoV-2 3CL Mpro, (A) RMSD values per residue (B) Trajectory RMSD (C) Radius of Gyration (D) B-factor per residue. SARS-CoV-2 3CL Mpro in the presence of Suramin, (A) RMSD values per residue (B) Trajectory RMSD (C) Radius of Gyration (D) B-factor per residue. SARS-CoV-2 3CL Mpro in the presence of 2S albumin, (A) RMSD values per residue (B) Trajectory RMSD (C) Radius of Gyration (D) B-factor per residue. SARS-CoV-2 3CL Mpro in the presence of flocculating, (A) RMSD values per residue (B) Trajectory RMSD (C) Radius of Gyration (D) B-factor per residue. [file Data_Sheet_1.PDF]

Supplementary materials

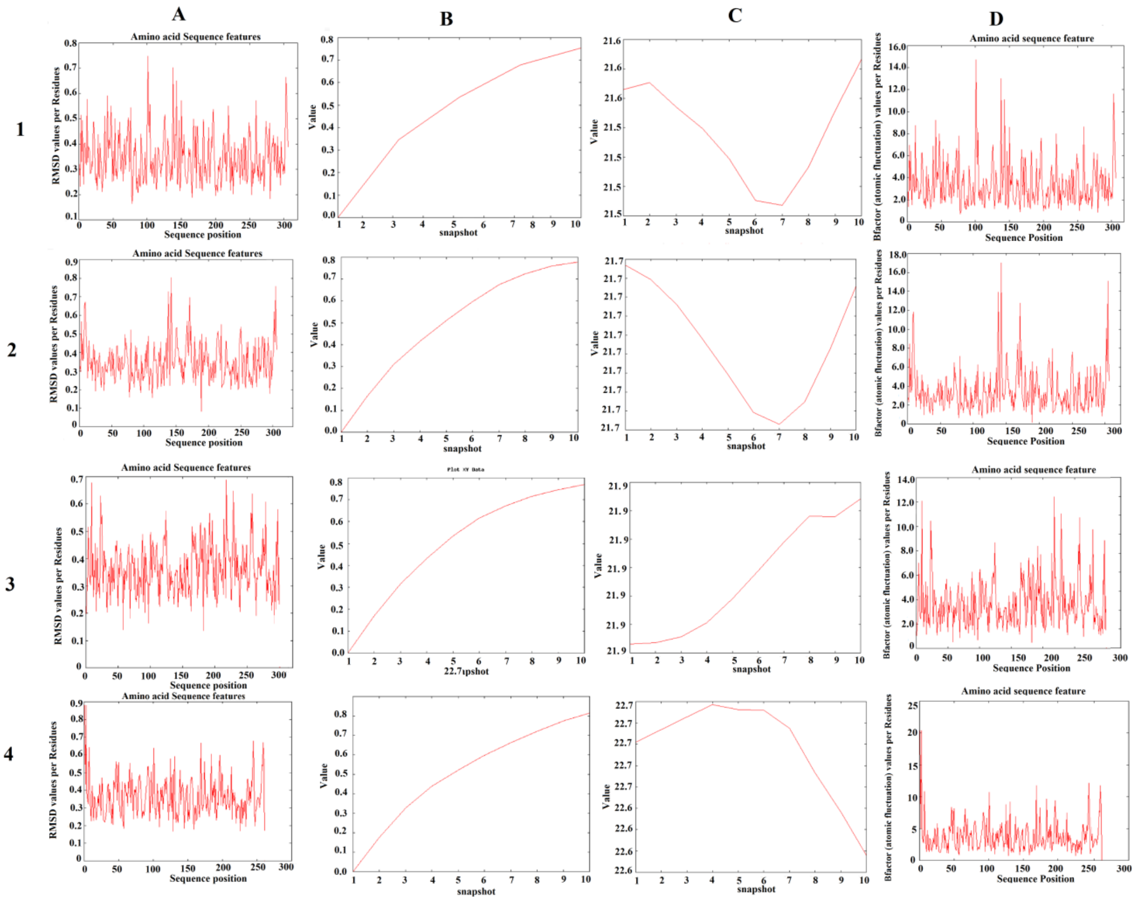

Figure S1: Molecular dynamic simulation of SARS-CoV-2 3CL M^{pro} and bound ligands

(1) SARS-CoV-2 3CL M^{pro}, **(A)** RMSD values per residue **(B)** Trajectory RMSD **(C)** Radius of Gyration **(D)** B-factor per residue

(2) SARS-CoV-2 3CL M^{pro} in the presence of Suramin, **(A)** RMSD values per residue **(B)** Trajectory RMSD **(C)** Radius of Gyration **(D)** B-factor per residue

(3) SARS-CoV-2 3CL M^{pro} in the presence of 2S albumin, **(A)** RMSD values per residue **((B)** Trajectory RMSD **(C)** Radius of Gyration **(D)** B-factor per residue

(4) SARS-CoV-2 3CL M^{pro} in the presence of flocculating, **(A)** RMSD values per residue **(B)** Trajectory RMSD **(C)** Radius of Gyration **(D)** B-factor per residue

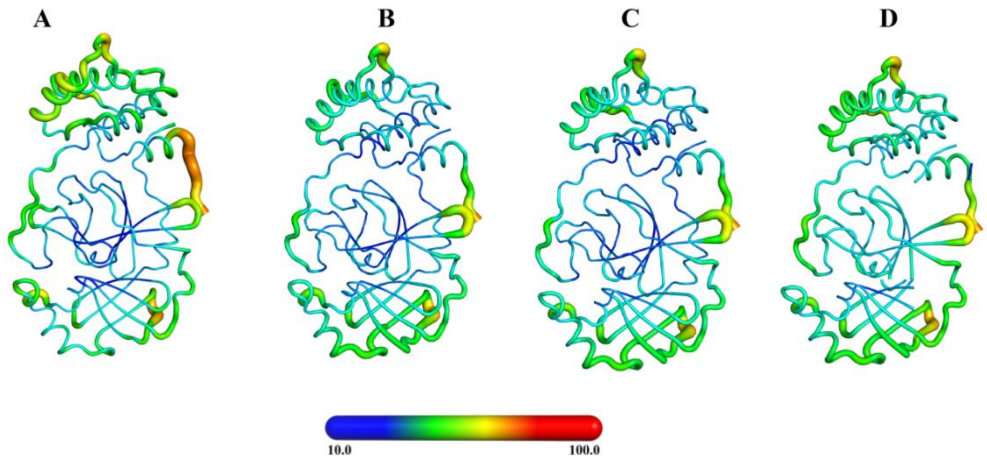

Figure S2: Temperature B-factor of SARS-CoV-2 3CL M^{pro} and bound ligands. **(A)** SARS-CoV-2 3CL M^{pro} alone **(B)** SARS-CoV-2 3CL M^{pro} with Suramin **(C)** SARS-CoV-2 3CL M^{pro} with 2S albumin **(D)** SARS-CoV-2 3CL M^{pro} with flocculating protein

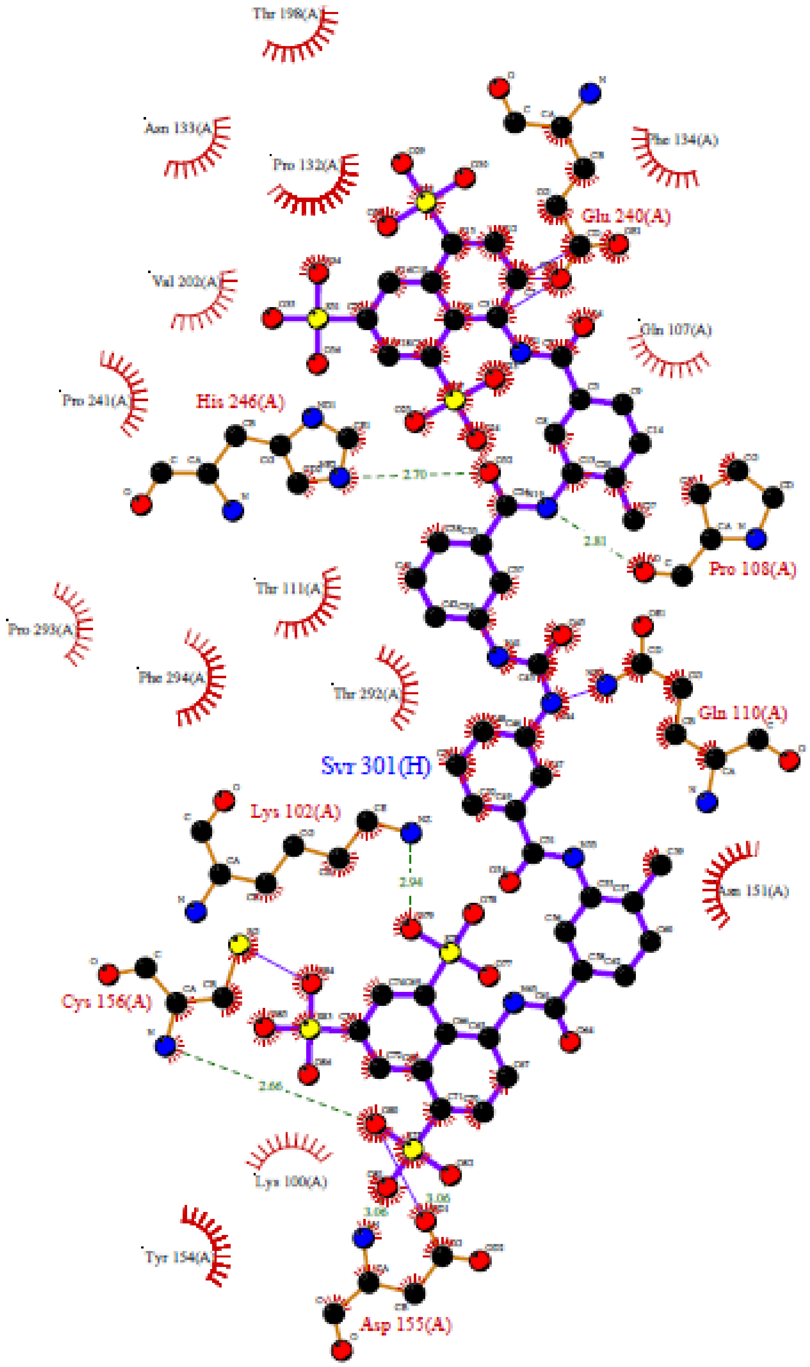

Figure S3: Hydrogen bonds and non-bonded contact (hydrophobic interactions) between 3CL M^{pro} and Suramin.

Table S1: List of protein-ligand interactions (3CL M^{pro} and Suramin)

Hydrogen bonds

	Atom 1: Atom no.	Atom 1: Atom name	Atom 1: Res name	Atom 1: Res no.	Atom 1: Chain		Atom 2: Atom no.	Atom 2: Atom name	Atom 2: Res name	Atom 2: Res no.	Atom 2: Chain	Distance
1.	796	NZ	LYS	102	A	-->	79	O79	SVR	301	H	2.94
2.	846	O	PRO	108	A	<--	19	N19	SVR	301	H	2.81
3.	1189	N	ASP	155	A	-->	81	O81	SVR	301	H	3.06
4.	1195	OD1	ASP	155	A	<--	82	O82	SVR	301	H	3.06
5.	1197	N	CYS	156	A	-->	80	O80	SVR	301	H	2.66
6.	1202	SG	CYS	156	A	-->	84	O84	SVR	301	H	2.17
7.	1936	NE2	HIS	246	A	-->	32	O32	SVR	301	H	2.70

Non-bonded contacts

	Atom 1: Atom no.	Atom 1: Atom name	Atom 1: Res name	Atom 1: Res no.	Atom 1: Chain		Atom 2: Atom no.	Atom 2: Atom name	Atom 2: Res name	Atom 2: Res no.	Atom 2: Chain	Distance
1.	773	CD	LYS	100	A	---	85	O85	SVR	301	H	3.67
2.	792	CB	LYS	102	A	---	84	O84	SVR	301	H	3.71
3.	794	CD	LYS	102	A	---	79	O79	SVR	301	H	3.75
4.	794	CD	LYS	102	A	---	84	O84	SVR	301	H	3.82
5.	795	CE	LYS	102	A	---	79	O79	SVR	301	H	3.88
6.	796	NZ	LYS	102	A	---	79	O79	SVR	301	H	2.94
7.	838	CB	GLN	107	A	---	27	C27	SVR	301	H	3.82
8.	846	O	PRO	108	A	---	8	C8	SVR	301	H	3.14
9.	846	O	PRO	108	A	---	13	C13	SVR	301	H	2.92
10.	846	O	PRO	108	A	---	19	N19	SVR	301	H	2.81
11.	846	O	PRO	108	A	---	20	C20	SVR	301	H	3.67
12.	847	CB	PRO	108	A	---	23	O23	SVR	301	H	3.84
13.	847	CB	PRO	108	A	---	24	O24	SVR	301	H	3.02
14.	848	CG	PRO	108	A	---	17	S17	SVR	301	H	3.64
15.	848	CG	PRO	108	A	---	23	O23	SVR	301	H	3.50
16.	848	CG	PRO	108	A	---	24	O24	SVR	301	H	3.12
17.	848	CG	PRO	108	A	---	25	O25	SVR	301	H	3.64
18.	855	CA	GLN	110	A	---	41	N41	SVR	301	H	3.53
19.	855	CA	GLN	110	A	---	43	C43	SVR	301	H	3.61
20.	855	CA	GLN	110	A	---	44	N44	SVR	301	H	3.58
21.	858	CB	GLN	110	A	---	41	N41	SVR	301	H	3.24
22.	858	CB	GLN	110	A	---	43	C43	SVR	301	H	3.23
23.	858	CB	GLN	110	A	---	44	N44	SVR	301	H	2.72
24.	858	CB	GLN	110	A	---	46	C46	SVR	301	H	3.53
25.	858	CB	GLN	110	A	---	48	C48	SVR	301	H	3.47
26.	859	CG	GLN	110	A	---	37	C37	SVR	301	H	3.66
27.	859	CG	GLN	110	A	---	39	C39	SVR	301	H	3.36
28.	859	CG	GLN	110	A	---	41	N41	SVR	301	H	2.24
29.	859	CG	GLN	110	A	---	43	C43	SVR	301	H	2.68
30.	859	CG	GLN	110	A	---	44	N44	SVR	301	H	2.39
31.	859	CG	GLN	110	A	---	45	O45	SVR	301	H	3.85
32.	859	CG	GLN	110	A	---	46	C46	SVR	301	H	3.61
33.	860	CD	GLN	110	A	---	41	N41	SVR	301	H	2.84
34.	860	CD	GLN	110	A	---	43	C43	SVR	301	H	2.97
35.	860	CD	GLN	110	A	---	44	N44	SVR	301	H	2.22
36.	860	CD	GLN	110	A	---	46	C46	SVR	301	H	3.24
37.	860	CD	GLN	110	A	---	48	C48	SVR	301	H	3.88
38.	862	OE1	GLN	110	A	---	41	N41	SVR	301	H	3.81
39.	862	OE1	GLN	110	A	---	44	N44	SVR	301	H	3.41
40.	861	NE2	GLN	110	A	---	41	N41	SVR	301	H	2.97
41.	861	NE2	GLN	110	A	---	43	C43	SVR	301	H	2.54
42.	861	NE2	GLN	110	A	---	44	N44	SVR	301	H	1.46
43.	861	NE2	GLN	110	A	---	45	O45	SVR	301	H	3.54
44.	861	NE2	GLN	110	A	---	46	C46	SVR	301	H	2.14
45.	861	NE2	GLN	110	A	---	47	C47	SVR	301	H	2.87
46.	861	NE2	GLN	110	A	---	48	C48	SVR	301	H	3.02
47.	863	N	THR	111	A	---	48	C48	SVR	301	H	3.68
48.	866	O	THR	111	A	---	48	C48	SVR	301	H	2.97
49.	866	O	THR	111	A	---	50	C50	SVR	301	H	2.88
50.	1019	CA	PRO	132	A	---	17	S17	SVR	301	H	3.68
51.	1019	CA	PRO	132	A	---	23	O23	SVR	301	H	3.70

52.	1019	CA	PRO	132	A	---	24	O24	SVR	301	H	2.86
53.	1020	C	PRO	132	A	---	11	C11	SVR	301	H	3.89
54.	1020	C	PRO	132	A	---	17	S17	SVR	301	H	3.77
55.	1020	C	PRO	132	A	---	18	C18	SVR	301	H	3.52
56.	1020	C	PRO	132	A	---	23	O23	SVR	301	H	3.29
57.	1020	C	PRO	132	A	---	24	O24	SVR	301	H	3.51
58.	1020	C	PRO	132	A	---	34	O34	SVR	301	H	3.81
59.	1021	O	PRO	132	A	---	11	C11	SVR	301	H	3.24
60.	1021	O	PRO	132	A	---	17	S17	SVR	301	H	3.02
61.	1021	O	PRO	132	A	---	18	C18	SVR	301	H	2.75
62.	1021	O	PRO	132	A	---	22	C22	SVR	301	H	3.75
63.	1021	O	PRO	132	A	---	23	O23	SVR	301	H	2.25
64.	1021	O	PRO	132	A	---	24	O24	SVR	301	H	3.22
65.	1021	O	PRO	132	A	---	34	O34	SVR	301	H	3.42
66.	1022	CB	PRO	132	A	---	6	C6	SVR	301	H	3.80
67.	1022	CB	PRO	132	A	---	11	C11	SVR	301	H	3.46
68.	1022	CB	PRO	132	A	---	17	S17	SVR	301	H	3.71
69.	1022	CB	PRO	132	A	---	18	C18	SVR	301	H	3.76
70.	1022	CB	PRO	132	A	---	24	O24	SVR	301	H	2.95
71.	1023	CG	PRO	132	A	---	24	O24	SVR	301	H	3.85
72.	1025	N	ASN	133	A	---	34	O34	SVR	301	H	3.73
73.	1026	CA	ASN	133	A	---	34	O34	SVR	301	H	3.31
74.	1029	CB	ASN	133	A	---	34	O34	SVR	301	H	3.46
75.	1042	CE2	PHE	134	A	---	23	O23	SVR	301	H	3.35
76.	1043	CZ	PHE	134	A	---	23	O23	SVR	301	H	3.66
77.	1158	CG	ASN	151	A	---	50	C50	SVR	301	H	3.04
78.	1158	CG	ASN	151	A	---	52	C52	SVR	301	H	3.38
79.	1160	OD1	ASN	151	A	---	50	C50	SVR	301	H	3.08
80.	1160	OD1	ASN	151	A	---	52	C52	SVR	301	H	3.50
81.	1159	ND2	ASN	151	A	---	48	C48	SVR	301	H	3.74
82.	1159	ND2	ASN	151	A	---	50	C50	SVR	301	H	2.84
83.	1159	ND2	ASN	151	A	---	52	C52	SVR	301	H	3.50
84.	1170	CA	ASP	153	A	---	63	N63	SVR	301	H	3.45
85.	1170	CA	ASP	153	A	---	65	C65	SVR	301	H	2.97
86.	1170	CA	ASP	153	A	---	66	C66	SVR	301	H	3.73
87.	1170	CA	ASP	153	A	---	67	C67	SVR	301	H	2.58
88.	1170	CA	ASP	153	A	---	70	C70	SVR	301	H	3.07
89.	1170	CA	ASP	153	A	---	71	C71	SVR	301	H	3.78
90.	1171	C	ASP	153	A	---	65	C65	SVR	301	H	3.50
91.	1171	C	ASP	153	A	---	67	C67	SVR	301	H	2.67
92.	1171	C	ASP	153	A	---	68	C68	SVR	301	H	3.84
93.	1171	C	ASP	153	A	---	70	C70	SVR	301	H	2.41
94.	1171	C	ASP	153	A	---	71	C71	SVR	301	H	3.09
95.	1171	C	ASP	153	A	---	75	S75	SVR	301	H	3.85
96.	1171	C	ASP	153	A	---	81	O81	SVR	301	H	3.79
97.	1172	O	ASP	153	A	---	65	C65	SVR	301	H	3.54
98.	1172	O	ASP	153	A	---	66	C66	SVR	301	H	3.65
99.	1172	O	ASP	153	A	---	67	C67	SVR	301	H	2.88
100.	1172	O	ASP	153	A	---	68	C68	SVR	301	H	3.06
101.	1172	O	ASP	153	A	---	70	C70	SVR	301	H	2.15
102.	1172	O	ASP	153	A	---	71	C71	SVR	301	H	2.27
103.	1172	O	ASP	153	A	---	75	S75	SVR	301	H	2.79
104.	1172	O	ASP	153	A	---	80	O80	SVR	301	H	2.82
105.	1172	O	ASP	153	A	---	81	O81	SVR	301	H	3.00
106.	1173	CB	ASP	153	A	---	61	C61	SVR	301	H	3.31
107.	1173	CB	ASP	153	A	---	63	N63	SVR	301	H	2.13
108.	1173	CB	ASP	153	A	---	64	O64	SVR	301	H	3.88
109.	1173	CB	ASP	153	A	---	65	C65	SVR	301	H	1.62
110.	1173	CB	ASP	153	A	---	66	C66	SVR	301	H	2.38
111.	1173	CB	ASP	153	A	---	67	C67	SVR	301	H	1.93
112.	1173	CB	ASP	153	A	---	68	C68	SVR	301	H	3.09
113.	1173	CB	ASP	153	A	---	69	C69	SVR	301	H	3.30
114.	1173	CB	ASP	153	A	---	70	C70	SVR	301	H	2.78
115.	1173	CB	ASP	153	A	---	71	C71	SVR	301	H	3.26
116.	1173	CB	ASP	153	A	---	73	S73	SVR	301	H	3.84
117.	1173	CB	ASP	153	A	---	78	O78	SVR	301	H	3.25
118.	1174	CG	ASP	153	A	---	61	C61	SVR	301	H	2.54
119.	1174	CG	ASP	153	A	---	63	N63	SVR	301	H	1.78
120.	1174	CG	ASP	153	A	---	64	O64	SVR	301	H	2.67
121.	1174	CG	ASP	153	A	---	65	C65	SVR	301	H	0.80
122.	1174	CG	ASP	153	A	---	66	C66	SVR	301	H	2.04

123.	1174	CG	ASP	153	A	---	67	C67	SVR	301	H	0.66
124.	1174	CG	ASP	153	A	---	68	C68	SVR	301	H	2.70
125.	1174	CG	ASP	153	A	---	69	C69	SVR	301	H	3.27
126.	1174	CG	ASP	153	A	---	70	C70	SVR	301	H	1.92
127.	1174	CG	ASP	153	A	---	71	C71	SVR	301	H	2.66
128.	1174	CG	ASP	153	A	---	78	O78	SVR	301	H	3.81
129.	1175	OD1	ASP	153	A	---	58	C58	SVR	301	H	3.44
130.	1175	OD1	ASP	153	A	---	61	C61	SVR	301	H	2.14
131.	1175	OD1	ASP	153	A	---	63	N63	SVR	301	H	2.04
132.	1175	OD1	ASP	153	A	---	64	O64	SVR	301	H	2.03
133.	1175	OD1	ASP	153	A	---	65	C65	SVR	301	H	1.83
134.	1175	OD1	ASP	153	A	---	66	C66	SVR	301	H	3.18
135.	1175	OD1	ASP	153	A	---	67	C67	SVR	301	H	1.22
136.	1175	OD1	ASP	153	A	---	68	C68	SVR	301	H	3.84
137.	1175	OD1	ASP	153	A	---	70	C70	SVR	301	H	2.49
138.	1175	OD1	ASP	153	A	---	71	C71	SVR	301	H	3.59
139.	1176	OD2	ASP	153	A	---	61	C61	SVR	301	H	3.11
140.	1176	OD2	ASP	153	A	---	63	N63	SVR	301	H	2.47
141.	1176	OD2	ASP	153	A	---	64	O64	SVR	301	H	2.98
142.	1176	OD2	ASP	153	A	---	65	C65	SVR	301	H	1.15
143.	1176	OD2	ASP	153	A	---	66	C66	SVR	301	H	1.41
144.	1176	OD2	ASP	153	A	---	67	C67	SVR	301	H	1.32
145.	1176	OD2	ASP	153	A	---	68	C68	SVR	301	H	1.72
146.	1176	OD2	ASP	153	A	---	69	C69	SVR	301	H	2.77
147.	1176	OD2	ASP	153	A	---	70	C70	SVR	301	H	1.69
148.	1176	OD2	ASP	153	A	---	71	C71	SVR	301	H	1.86
149.	1176	OD2	ASP	153	A	---	72	C72	SVR	301	H	3.06
150.	1176	OD2	ASP	153	A	---	73	S73	SVR	301	H	3.84
151.	1176	OD2	ASP	153	A	---	74	C74	SVR	301	H	3.74
152.	1176	OD2	ASP	153	A	---	75	S75	SVR	301	H	3.43
153.	1176	OD2	ASP	153	A	---	76	C76	SVR	301	H	3.87
154.	1177	N	TYR	154	A	---	67	C67	SVR	301	H	3.41
155.	1177	N	TYR	154	A	---	70	C70	SVR	301	H	3.09
156.	1178	CA	TYR	154	A	---	70	C70	SVR	301	H	3.67
157.	1178	CA	TYR	154	A	---	81	O81	SVR	301	H	3.46
158.	1179	C	TYR	154	A	---	81	O81	SVR	301	H	3.74
159.	1181	CB	TYR	154	A	---	70	C70	SVR	301	H	3.17
160.	1181	CB	TYR	154	A	---	71	C71	SVR	301	H	3.81
161.	1181	CB	TYR	154	A	---	75	S75	SVR	301	H	3.53
162.	1181	CB	TYR	154	A	---	81	O81	SVR	301	H	2.32
163.	1182	CG	TYR	154	A	---	81	O81	SVR	301	H	2.82
164.	1184	CD2	TYR	154	A	---	81	O81	SVR	301	H	2.74
165.	1189	N	ASP	155	A	---	75	S75	SVR	301	H	3.63
166.	1189	N	ASP	155	A	---	80	O80	SVR	301	H	3.11
167.	1189	N	ASP	155	A	---	81	O81	SVR	301	H	3.06
168.	1190	CA	ASP	155	A	---	80	O80	SVR	301	H	3.48
169.	1191	C	ASP	155	A	---	80	O80	SVR	301	H	3.23
170.	1193	CB	ASP	155	A	---	80	O80	SVR	301	H	3.73
171.	1194	CG	ASP	155	A	---	75	S75	SVR	301	H	3.66
172.	1194	CG	ASP	155	A	---	80	O80	SVR	301	H	2.83
173.	1194	CG	ASP	155	A	---	81	O81	SVR	301	H	3.44
174.	1195	OD1	ASP	155	A	---	71	C71	SVR	301	H	3.85
175.	1195	OD1	ASP	155	A	---	75	S75	SVR	301	H	2.41
176.	1195	OD1	ASP	155	A	---	80	O80	SVR	301	H	1.75
177.	1195	OD1	ASP	155	A	---	81	O81	SVR	301	H	2.32
178.	1195	OD1	ASP	155	A	---	82	O82	SVR	301	H	3.06
179.	1196	OD2	ASP	155	A	---	80	O80	SVR	301	H	3.64
180.	1197	N	CYS	156	A	---	75	S75	SVR	301	H	3.90
181.	1197	N	CYS	156	A	---	80	O80	SVR	301	H	2.66
182.	1198	CA	CYS	156	A	---	80	O80	SVR	301	H	3.32
183.	1201	CB	CYS	156	A	---	68	C68	SVR	301	H	3.48
184.	1201	CB	CYS	156	A	---	72	C72	SVR	301	H	2.95
185.	1201	CB	CYS	156	A	---	76	C76	SVR	301	H	3.32
186.	1201	CB	CYS	156	A	---	80	O80	SVR	301	H	2.94
187.	1201	CB	CYS	156	A	---	83	S83	SVR	301	H	3.76
188.	1201	CB	CYS	156	A	---	84	O84	SVR	301	H	3.37
189.	1202	SG	CYS	156	A	---	72	C72	SVR	301	H	3.64
190.	1202	SG	CYS	156	A	---	74	C74	SVR	301	H	3.83
191.	1202	SG	CYS	156	A	---	76	C76	SVR	301	H	3.27
192.	1202	SG	CYS	156	A	---	83	S83	SVR	301	H	3.19
193.	1202	SG	CYS	156	A	---	84	O84	SVR	301	H	2.17

194.	1202	SG	CYS	156	A	---	85	O85	SVR	301	H	3.84
195.	1215	OG	SER	158	A	---	78	O78	SVR	301	H	3.79
196.	1215	OG	SER	158	A	---	79	O79	SVR	301	H	3.84
197.	1506	N	THR	196	A	---	35	O35	SVR	301	H	3.80
198.	1509	O	THR	196	A	---	34	O34	SVR	301	H	3.53
199.	1509	O	THR	196	A	---	35	O35	SVR	301	H	3.43
200.	1525	CB	THR	198	A	---	28	O28	SVR	301	H	3.60
201.	1527	OG1	THR	198	A	---	28	O28	SVR	301	H	3.67
202.	1526	CG2	THR	198	A	---	21	S21	SVR	301	H	3.71
203.	1526	CG2	THR	198	A	---	28	O28	SVR	301	H	2.49
204.	1555	CG1	VAL	202	A	---	38	C38	SVR	301	H	3.53
205.	1870	O	TYR	239	A	---	30	O30	SVR	301	H	3.87
206.	1880	CA	GLU	240	A	---	7	C7	SVR	301	H	3.87
207.	1880	CA	GLU	240	A	---	12	C12	SVR	301	H	3.67
208.	1883	CB	GLU	240	A	---	4	O4	SVR	301	H	3.26
209.	1883	CB	GLU	240	A	---	7	C7	SVR	301	H	3.53
210.	1883	CB	GLU	240	A	---	12	C12	SVR	301	H	3.86
211.	1884	CG	GLU	240	A	---	1	N1	SVR	301	H	3.87
212.	1884	CG	GLU	240	A	---	2	C2	SVR	301	H	3.48
213.	1884	CG	GLU	240	A	---	3	C3	SVR	301	H	3.53
214.	1884	CG	GLU	240	A	---	4	O4	SVR	301	H	2.48
215.	1884	CG	GLU	240	A	---	7	C7	SVR	301	H	3.04
216.	1884	CG	GLU	240	A	---	12	C12	SVR	301	H	3.47
217.	1885	CD	GLU	240	A	---	1	N1	SVR	301	H	2.90
218.	1885	CD	GLU	240	A	---	2	C2	SVR	301	H	3.05
219.	1885	CD	GLU	240	A	---	3	C3	SVR	301	H	2.17
220.	1885	CD	GLU	240	A	---	4	O4	SVR	301	H	2.53
221.	1885	CD	GLU	240	A	---	6	C6	SVR	301	H	2.95
222.	1885	CD	GLU	240	A	---	7	C7	SVR	301	H	1.69
223.	1885	CD	GLU	240	A	---	10	C10	SVR	301	H	3.22
224.	1885	CD	GLU	240	A	---	12	C12	SVR	301	H	2.16
225.	1885	CD	GLU	240	A	---	15	C15	SVR	301	H	2.90
226.	1886	OE1	GLU	240	A	---	1	N1	SVR	301	H	2.17
227.	1886	OE1	GLU	240	A	---	2	C2	SVR	301	H	2.53
228.	1886	OE1	GLU	240	A	---	3	C3	SVR	301	H	1.61
229.	1886	OE1	GLU	240	A	---	4	O4	SVR	301	H	2.43
230.	1886	OE1	GLU	240	A	---	6	C6	SVR	301	H	2.83
231.	1886	OE1	GLU	240	A	---	7	C7	SVR	301	H	0.81
232.	1886	OE1	GLU	240	A	---	10	C10	SVR	301	H	3.33
233.	1886	OE1	GLU	240	A	---	12	C12	SVR	301	H	1.96
234.	1886	OE1	GLU	240	A	---	15	C15	SVR	301	H	3.01
235.	1887	OE2	GLU	240	A	---	1	N1	SVR	301	H	3.45
236.	1887	OE2	GLU	240	A	---	2	C2	SVR	301	H	3.87
237.	1887	OE2	GLU	240	A	---	3	C3	SVR	301	H	2.38
238.	1887	OE2	GLU	240	A	---	4	O4	SVR	301	H	3.47
239.	1887	OE2	GLU	240	A	---	6	C6	SVR	301	H	2.49
240.	1887	OE2	GLU	240	A	---	7	C7	SVR	301	H	2.23
241.	1887	OE2	GLU	240	A	---	10	C10	SVR	301	H	2.38
242.	1887	OE2	GLU	240	A	---	11	C11	SVR	301	H	3.61
243.	1887	OE2	GLU	240	A	---	12	C12	SVR	301	H	2.15
244.	1887	OE2	GLU	240	A	---	15	C15	SVR	301	H	2.23
245.	1887	OE2	GLU	240	A	---	16	C16	SVR	301	H	3.44
246.	1887	OE2	GLU	240	A	---	21	S21	SVR	301	H	3.40
247.	1887	OE2	GLU	240	A	---	28	O28	SVR	301	H	3.65
248.	1887	OE2	GLU	240	A	---	30	O30	SVR	301	H	3.87
249.	1894	CD	PRO	241	A	---	7	C7	SVR	301	H	3.59
250.	1894	CD	PRO	241	A	---	12	C12	SVR	301	H	3.25
251.	1933	CD2	HIS	246	A	---	32	O32	SVR	301	H	3.27
252.	1935	CE1	HIS	246	A	---	32	O32	SVR	301	H	3.84
253.	1936	NE2	HIS	246	A	---	26	C26	SVR	301	H	3.84
254.	1936	NE2	HIS	246	A	---	32	O32	SVR	301	H	2.70
255.	2268	CB	THR	292	A	---	45	O45	SVR	301	H	3.69
256.	2270	OG1	THR	292	A	---	45	O45	SVR	301	H	3.82
257.	2269	CG2	THR	292	A	---	45	O45	SVR	301	H	3.51
258.	2277	CD	PRO	293	A	---	40	C40	SVR	301	H	3.88
259.	2284	CD1	PHE	294	A	---	59	C59	SVR	301	H	3.43
260.	2285	CD2	PHE	294	A	---	45	O45	SVR	301	H	3.88
261.	2286	CE1	PHE	294	A	---	59	C59	SVR	301	H	3.11
262.	2287	CE2	PHE	294	A	---	59	C59	SVR	301	H	3.82
263.	2288	CZ	PHE	294	A	---	59	C59	SVR	301	H	3.32

Number of hydrogen bonds: 7
Number of non-bonded contacts: 263

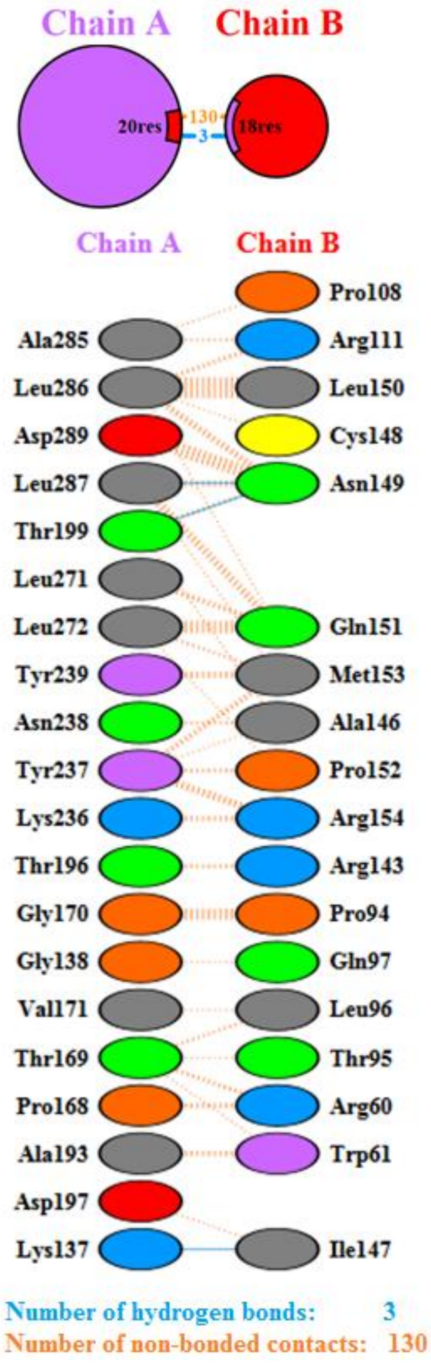

Figure S4: Hydrogen bonds and non-bonded contact (hydrophobic interactions) between 3CL M^{pro} and 2s albumin.

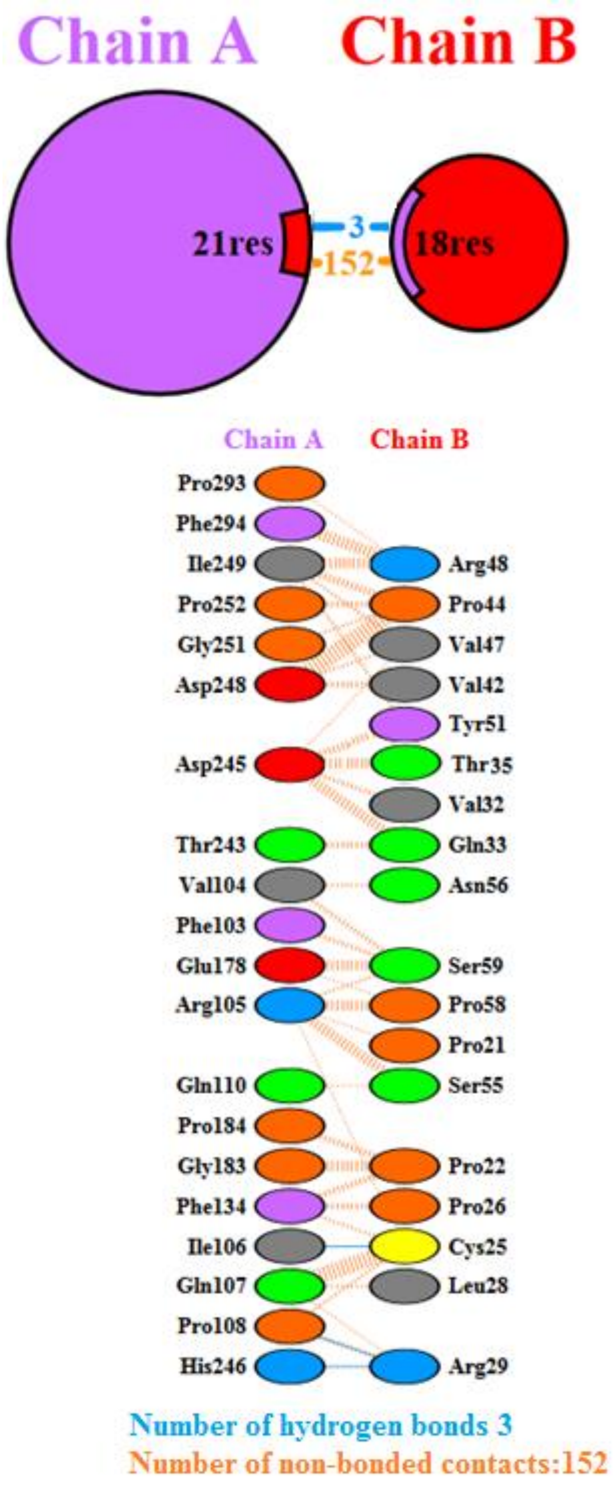

Figure S5: Hydrogen bonds and non-bonded contact (hydrophobic interactions) between 3CL M^{pro} and flocculating protein.

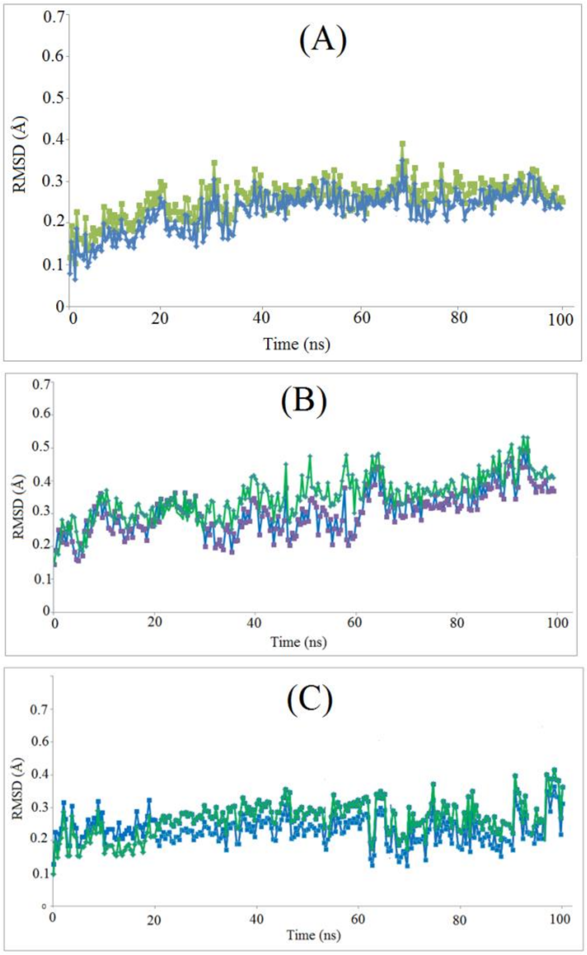

Figure S6: RMSD vs Time graph for 100 ns MD simulation of 3CLM^{pro} (green) with **(A)** Suramin **(B)** 2S albumin **(C)** Flocculating protein (blue)

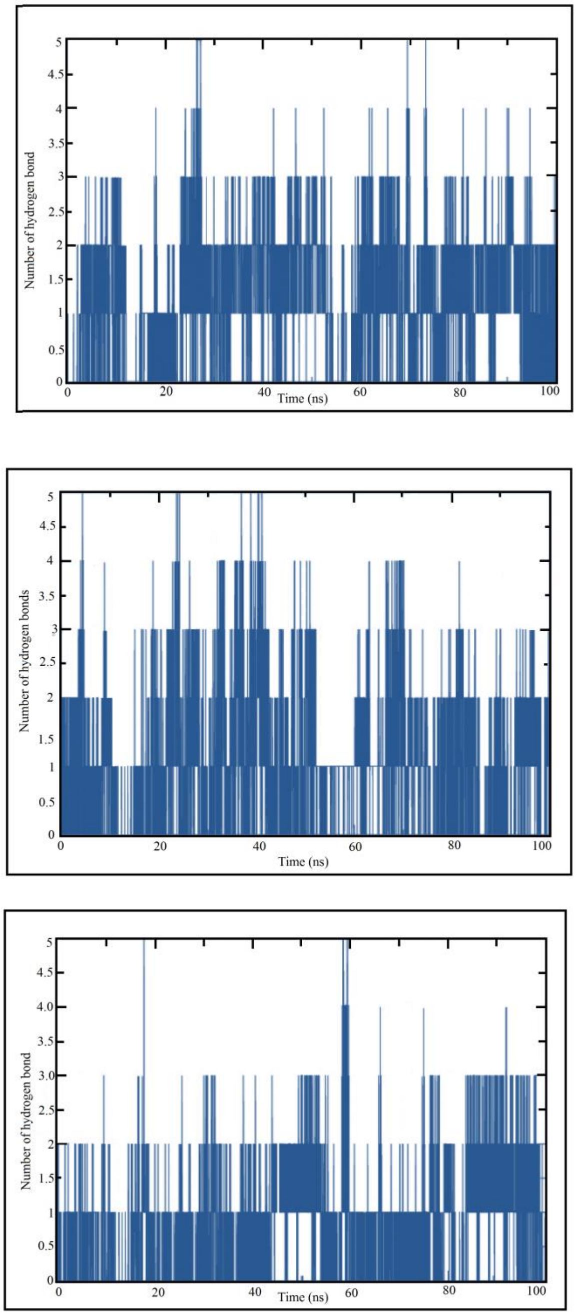

(A)

(B)

(C)

Figure S7: Graphs of hydrogen bonds plotted vs time for 100 ns range simulations of 3CLM^{pro} and (A) Suramin (B) 2S albumin (C) Flocculating protein
